# The establishment of the general microexpression recognition ability and its relevant brain activity

**DOI:** 10.3389/fnhum.2022.894702

**Published:** 2022-12-07

**Authors:** Jianxin Zhang, Ming Yin, Deming Shu, Dianzhi Liu

**Affiliations:** ^1^Jiangsu Province Engineering Research Center of Microexpression Intelligent Sensing and Security Prevention and Control, Nanjing, China; ^2^School of Education, Jiangnan University, Wuxi, China; ^3^Jiangsu Police Institute, Nanjing, China; ^4^School of Education, Soochow University, Soochow, China

**Keywords:** GMERA, PREMERT, three-layer hierarchical structure, ALFFs, overlap

## Abstract

Microexpressions are very transitory expressions lasting about 1/25∼1/2 s, which can reveal people’s true emotions they try to hide or suppress. The PREMERT (pseudorandom ecological microexpression recognition test) could test the individual’s microexpression recognition ability with six microexpression *M*s (the mean of accuracy rates of a microexpression type under six expression backgrounds), and six microexpression *SD*s (the standard deviation of accuracy rates of this microexpression type under six expression backgrounds), but it and other studies did not explore the general microexpression recognition ability (the GMERA) or could not test the GMERA effectively. Therefore, the current study put forward and established the GMERA with the behavioral data of the PREMERT. The spontaneous brain activity in the resting state is a stable index to measure individual cognitive characteristics. Therefore, the current study explored the relevant resting-state brain activity of the GMERA indicators to prove that GMERA is an individual cognitive characteristic from brain mechanisms with the neuroimaging data of the PREMERT. The results showed that (1) there was a three-layer hierarchical structure in human microexpression recognition ability: The GMERA (the highest layer); recognition of a type of microexpression under different expression backgrounds (the second layer); and recognition of a certain microexpression under a certain expression background (the third layer). A common factor GMERA was extracted from the six microexpression types recognition in PREMERT. Four indicators of the GMERA were calculated from six microexpression *M*s and six microexpression *SD*s, such as GMERAL (level of GMERA), GMERAF (fluctuation of GMERA), GMERAB (background effect of GMERA), and GMERABF (fluctuation of GMERAB), which had good parallel-forms reliability, calibration validity, and ecological validity. The GMERA provided a concise and comprehensive overview of the individual’s microexpression recognition ability. The PREMERT was proved as a good test to measure the GMERA. (2) ALFFs (the amplitude of low-frequency fluctuations) in both eyes-closed and eyes-opened resting-states and ALFFs-difference could predict the four indicators of the GMERA. The relevant resting-state brain areas were some areas of the expression recognition network, the microexpression consciousness and attention network, and the motor network for the change from expression backgrounds to microexpression. (3) The relevant brain areas of the GMERA and different types of microexpression recognition belonged to the three cognitive processes, but the relevant brain areas of the GMERA were the “higher-order” areas to be more concise and critical than those of different types of microexpression recognition.

## Introduction

Microexpressions are very transitory expressions lasting about 1/25∼1/2 s, which can reveal people’s true emotions they try to hide or suppress (Ekman and Friesen, 1975; Porter et al., 2012). Even if liars deliberately use the dissemble strategy, they still cannot prevent the disclosure of the true expression, thus producing microexpressions. Therefore, microexpressions can be used as an important tool to detect real emotions and lies (Shen et al., 2017). Microexpression recognition ability is the ability of a person or a machine to recognize another person’s microexpressions. In the current study, we only explore the human microexpression recognition ability. A microexpression recognition test with good simplicity, reliability, validity, and ecological validity is very important, which can quickly and effectively measure a person’s microexpression recognition ability to screen and cultivate excellent talents. Furthermore, some representative indicators are needed, which should measure people’s microexpression recognition ability stably, effectively, succinctly, and comprehensively. Therefore, they should have good reliability and validity, have as few indicators as possible, and can measure the overall recognition ability of different microexpressions under different expression backgrounds, such as sadness, fear, disgust, surprise, and happiness, and microexpressions under sadness, fear, disgust, neutral, surprise, and happiness expression backgrounds (Zhang et al., 2017, 2020b; Yin et al., 2019, 2020). It is obvious that the representative indicators must be based on good tests.

Spearman (1928) proposed that human intelligence includes two factors: One is the general intelligence factor (GIF), which is derived from innate inheritance and manifested in general activities, indicating the level of individual ability. The other is a specific factor, which is the ability of an individual to perform a special activity that differs from others. Vernon (1966) proposed that the structure of intelligence is arranged in hierarchies: The highest is the GIF; the second layer is two big factors, namely, speech and education factor, and operation and mechanical factor; the third layer is five small factors, including speech, quantity, mechanical information, spatial ability, and manual operation; and the fourth layer is special factors. The general intelligence test measures the GIF. Darwin (1871) and Galton (1887) proposed that humans may be under evolutionary selection pressure to form the general personality factor (GPF) in the same way that the GIF is formed. Musek (2007) adopted stepwise higher-order factor analyses to conduct exploratory factor analysis on multiple Big Five personality questionnaires and obtained big two factors above the big five dimensions, and the GPF was extracted based on big two factors, which explained more than 60% of the common variation in sophomore factors.

**FIGURE 1:**
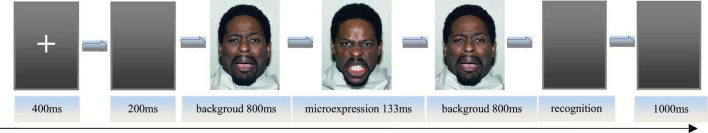
The picture of the experiment procedure from Zhang et al. (2020b). These images are licensed by the copyright owners, Tottenham et al. (2009) and Zhang et al. (2020b).

Drawing on the GIF (Spearman, 1928; Vernon, 1966) and GPF (Musek, 2007; Wu, 2017; Wu et al., 2021), the current study put forward the concept of the general microexpression recognition ability (GMERA). We assumed that human microexpression recognition ability has a three-layer hierarchical structure: The highest layer is the GMERA, which may be derived from innate heredity and influenced by learning, and manifests the level of individual microexpression recognition ability (the current study); the second layer is the individual’s ability to recognize a type of microexpression under different expression backgrounds, for example, anger microexpression under sadness, fear, disgust, neutral, surprise, and happiness expression backgrounds (Yin et al., 2020; Zhang et al., 2020b); and the third layer is an individual’s ability to recognize a certain microexpression under a certain expression background, for example, anger microexpression under sadness expression background (please refer to Figure 1, Zhang et al., 2017; Zhu et al., 2017; Yin et al., 2019). The GMERA can be obtained by extracting a common factor from various kinds of microexpressions recognition abilities. Its tests and indicators should meet the above requirements.

Matsumoto et al. (2000) developed the Japanese and Caucasian Brief Affect Recognition Test (JACBART, classical microexpression recognition) to measure the microexpression recognition ability (Hall and Matsumoto, 2004; Matsumoto and Hwang, 2011). It only used the neutral expressions backgrounds before and after a brief microexpression to hide it but did not examine the influence of backgrounds with emotional expressions. The real microexpressions are embedded in the backgrounds of various expressions. Therefore, the ecological validity of JACBART is not good enough to test the GMERA.

Zhang et al. (2017) examined the recognition characteristics of six basic microexpression types (sadness, fear, anger, disgust, surprise, and happiness) under seven basic expression backgrounds (the six basic expressions and neutral) to establish an ecological microexpression recognition test (EMERT), and obtained the recognition accuracy rates and background effects of the six microexpression types. Zhu et al. (2017) used a simplified edition of EMERT to find the microexpression recognition difference between depressive patients and normal people. Yin et al. (2019) extended EMERT to WEMERT (weak). Although the series of EMERTs improved the ecological validity of the microexpression recognition test, they used a between-subjects Latin Square block design for backgrounds, which made the participants perform different EMERTs. The two participants’ scores could not be compared strictly because the exercise effect and fatigue effect between them were different. They cannot be used to test the GMERA. They did not come up with the concept and operational definition of the GMERA too. Therefore, Zhang et al. (2020b) for the first time used the within-subject pseudorandom design backgrounds to improve EMERT to PREMERT (pseudorandom EMERT). Each participant took the same test and got comparable test scores.

The spontaneous brain activity in the resting state is a stable index to measure individual cognitive characteristics (Liu et al., 2017), such as emotional ability (Heuvel and Pol, 2010; Li X., 2015; Xue et al., 2015), intelligence (Langer et al., 2012; Zhang, 2021), creativity (Li et al., 2016; Jiang et al., 2018), music skills (Leipold et al., 2021; Long, 2022), and personality (Adelstein et al., 2011; Wang, 2016). A kind of inherited neural activation pattern indicated by specific resting-state activations could predict microexpression perception performance (Yin et al., 2020; Zhang et al., 2020b). Yin et al. (2020) used eyes-closed and eyes-opened resting-state fMRI to find that in EMERT, the ALFFs-relevant brain areas (brain areas whose ALFFs were related to the microexpression recognition) of the six microexpression types recognition were some frontal lobes, insula, cingulate cortex, hippocampus, parietal lobe, caudate nucleus, thalamus, amygdala, occipital lobe, fusiform, temporal lobe, cerebellum, and vermis, and the ALFFs-relevant brain areas of background effects were some frontal lobes, insula, cingulate cortex, cuneus, amygdala, fusiform, occipital lobe, parietal lobe, precuneus, caudate lobe, putamen lobe, thalamus, temporal lobe, cerebellum, and vermis. Zhang et al. (2020b) found that in PREMERT, the ALFFs-relevant brain areas of the six microexpression types recognition were some frontal lobes, insula, cingulate cortex, hippocampus, amygdala, fusiform gyrus, parietal lobe, caudate nucleus, precuneus, thalamus, putamen, temporal lobe, and cerebellum, and the ALFFs-relevant brain areas of background effects were some frontal lobes, central anterior gyrus, supplementary motor area, insula, hippocampus, amygdala, cuneus, occipital lobe, fusiform gyrus, parietal lobe, caudate nucleus, pallidum, putamen, thalamus, temporal lobe, and cerebellum. The PREMERT can be used to test the GMERA. Unfortunately, the study only explored the recognition of the six microexpression types, but neither put forward nor calculated the GMERA, thus, they did not explore the inherited neural activation pattern of the GMERA. The relevant brain areas of the GMERA should be the “higher-order” areas compared to the brain areas related to different types of microexpression recognition.

Therefore, the current study puts forward the concept of the GMERA, used exploratory factor analysis to extract the common factor from the six microexpression types’ recognition scores to prove it, and calculated the average of the six microexpression types’ recognition scores as its operational definition. Then, the ALFFs-relevant brain areas of the indicators of the GMERA were explored to prove that GMERA is an individual cognitive characteristic of brain mechanisms. The overlap and difference between the relevant brain areas of the GMERA and different types of microexpression recognition were analyzed.

## Materials and methods

### Participants

The current study used the data of Zhang et al. (2020b) to make a new analysis. Fifty-three college students of Southwest University in China participated in the study. The number of male and female participants was 24 and 29, respectively. The age M ± SD = 21.60 ± 2.39. They were all right-handed with normal or corrected-to-normal eyesight and without color blindness. They had similar Chinese college entrance examination scores. They had never taken part in microexpression recognition experiments before and were not engaged in investigation, interrogation, lie detection, etc., thus, they had no training in lie detection, microexpression recognition, or other detection skills. In addition, it was explored which brain activity differences would influence differences in microexpression recognition ability. Therefore, individual differences were not interfering factors in the current study. All the participants met the criteria for functional magnetic resonance imaging (fMRI) scanning, namely, they had no metal implants, were not claustrophobic, and had a head size compatible with the head coil. They all volunteered and could quit at any time. Each participant completed an informed consent form before the experiments. They got corresponding rewards after completing the experiments. The experiments were in accordance with the ethical guidelines of the Declaration of Helsinki and were approved by the Scientific Review Committee of the Faculty of Psychology, Southwest University, China.

### Experimental apparatus and materials

Seven kinds of basic expression opened-mouth pictures of eight models (four men and four women, including white, black, and yellow people) from the NimStim face expression database (Tottenham et al., 2009) were used as the backgrounds, namely, neutral, sadness, fear, anger, disgust, surprise, and happiness. Except for neutral expressions, the other six kinds of expressions were used as microexpressions. The pixels of all images were modified to be 338 × 434 with a gray background (GRB: 127, 127, and 127) (Zhang et al., 2017). The Lenovo ThinkPad T410i notebook computer and 14.1-inch LCD screen, which had 1,280 × 800 resolution and 60 Hz of refresh rate, were used to do the experiments. E-prime 2.0 was used to compile the experimental procedure.

### Experimental design and procedures

The experiment was 7 (expression backgrounds: neutral vs. sadness vs. fear vs. anger vs. disgust vs. surprise vs. happiness) × 6 (microexpression types: sadness vs. fear vs. anger vs. disgust vs. surprise vs. happiness) within-subject design. As there were seven expression backgrounds, in order to balance the sequential effect, the within-subject pseudorandom design for backgrounds was used in the current study rather than the Latin Square block design for backgrounds in EMERT (Zhang et al., 2017; Yin et al., 2020), and the within-subject pseudorandom design for microexpression types was also used in the current study as in EMERT.

Participants were 70 cm away from the screen. On the computer keyboard, six keys of SDF-JKL corresponded with sadness, fear, anger, disgust, surprise, and happiness. First, the center of the screen would show the “+” for 400 ms; second, the empty screen lasted 200 ms; then, the front expression background was presented for 800 ms, after which the microexpression image would appear for 133 ms, followed by 800 ms of back expression background (Matsumoto et al., 2000; Zhang et al., 2017). The front and back expression backgrounds and microexpressions were of the same model’s face and the front and back expression backgrounds were the same. Participants needed to try to identify the briefly presented microexpression between the front and back expression backgrounds. The participants were asked to press a key according to the microexpression they saw as accurately as possible instead of as soon as possible (no time limit). After the participants pressed the key, an empty screen would show for 1,000 ms. Then, the fixation point “+” was presented for 400 ms, and the next trial started. The experiment procedure is shown in Figure 1. For a detailed procedure, please see Zhang et al. (2020b).

A month before the PREMERT, all the participants lay in the fMRI scanner to undergo structural scanning, eyes-closed and eyes-opened resting-states scans. About a week later, the participants needed to do two EMERT measurements, such as EMERT1 and EMERT2. Because Zhang et al. (2017) and Yin et al. (2020) proved that EMERT had good reliability and validity, we used the correlation between PREMERT and EMERT as the parallel-forms reliability and criterion validity of the GMERA.

### Behavioral data collection and analysis

The mean of accuracy rates of a microexpression type under six expression backgrounds (except the same expression background as the microexpression, because in that case, it was a normal expression rather than a microexpression) was used as the index of this microexpression type recognition, and it was abbreviated as microexpression *M*. The standard deviation of accuracy rates of this microexpression type under six expression backgrounds (except the same expression background as the microexpression) was used as the background effect index of this microexpression type recognition (Zhang et al., 2017, 2020b; Yin et al., 2019, 2020), and it was abbreviated as microexpression *SD*. A single sample *t*-test was made for each microexpression *M* with random level 1/6 to find whether the participants could recognize the microexpression than random. A single sample *t*-test was made for each microexpression SD with random level 0 to find whether the background effect existed.

Exploratory factor analysis was conducted on the six microexpression *M*s to prove whether there was a common factor GEMRA. The principal axis factor decomposition method was adopted to extract factors based on eigen values greater than 1. The maximum convergence iterations were less than 100 times. On the premise of the existence of a common factor GEMRA, four indicators of GEMRA were calculated. The average of the six microexpression *M*s was calculated as the operational definition of GMERAL (level of GMERA). The standard deviation of the six microexpression *M*s was calculated as the operational definition of GMERAF (fluctuation of GMERA). The average of the six microexpression *SD*s was calculated as the operational definition of GMERAB (background effect of GMERA). The standard deviation of the six microexpression *SD*s was calculated as the operational definition of GMERABF (fluctuation of GMERAB). A single sample *t*-test was made for GMERAL with random level 1/6 to find whether the participants could recognize the microexpression than random. A single sample *t*-test was made for GMERAF, GMERAB, and GMERABF with random level 0 to find whether the fluctuation of GMERA existed.

The Pearson correlation was made between the four indicators of GEMRA in PREMERT and the corresponding indicators in EMERT1, EMERT2, JACBART, and expression to get the parallel-forms reliability, criterion validity, and ecological validity of the GMERA.

### Resting-state data collection and analysis

The participants were instructed that the fMRI was not harmful and that they should get enough sleep the night before the scans and take it easy during the scans. They lay in the fMRI scanner to undergo structural scanning, eyes-closed and eyes-opened (look at the cross naturally without great concentration) resting-states scans, each of which was 8 min. They were instructed not to think about anything, and if they could not help it, then not to think about certain things (Li et al., 2016; Liu et al., 2017; Jiang et al., 2018; Zhang et al., 2020a; Zhang and Liu, 2021).

The fMRI data were collected using a Siemens 3.0 T magnetic resonance imaging scanner and an 8-channel phased front head coil. Eyes-closed and eyes-opened resting-state imaging used gradient echo (GRE) single-excitation echo-planar imaging (EPI). Scan parameters were as follows: The phase encoding direction was R/L, TR = 2,000 ms, TE = 30 ms, FA = 90°, FOV = 220 mm × 220 mm, matrix size = 64 × 64, depth = 3 mm, planar resolution = 3.44 mm × 3.44 mm, interval scanning, 33 layers, layer spacing = 0.6 mm, total 240 layers, 8 min. Structural imaging used a 3D TlWI (MP-RAGE) sequence with sagittal scans. Scan parameters were as follows: TR = 2,600 ms, TE = 3.02 ms, FA = 8°, no interval, FOV = 256 mm × 256 mm, matrix size = 256 mm × 256 mm, total 176 layers. All the participants first received the structural scan, then half received the eyes-closed and eyes-opened resting-state scans, and half received the eyes-opened and eyes-closed resting-state scans.

Pretreatment and analysis of resting-state data used DPARSF 3.0 Advanced Edition Calculate (Yan et al., 2016) in Original Space (Warp by DARTEL), following standard procedures: (1) Conversion of raw DICOM-format data to NIFTI format. To allow for signal stabilization of the image, the first 10 TR images were removed, after which time layer correction (slice timing) and head movement correction (realignment, adopting Friston 24) were conducted. If head movement greater than 2 mm occurred during the resting state, the data were deleted. (2) The new segment + DARTEL was used to split the structural T1 data without standardization, and register the T1 split data directly to the resting-state functional images. Before registration of structural and functional data, the AC-PC line of each participant’s T1 image and the resting-state function were registered, and then automatic registration was applied. Therefore, the resting-state analysis took place in the original T1 space. (3) Head motion, linear drift, white matter, and cerebrospinal fluid *via* regression were adjusted for. (4) Low-frequency fluctuations ALFFs (filter range: 0.01–0.1 Hz) were calculated. (5) The resting-state function was registered to the standard MNI space (normalized by DARTEL), using Bounding Box [−90, −126, −72; 90, 90, 108] and a 3 mm × 3 mm × 3 mm voxel size, with 4 mm × 4 mm × 4 mm full width at half maximum (FWHM) smoothing.

REST1.8 (Song et al., 2011) was first used to extract the amplitude of low-frequency fluctuations (ALFFs, Zang et al., 2007) during resting-states in 116 Anatomical Automatic Labeling (AAL) brain areas. ALFFs were normalized to mALFFs. Second, SPSS19.0 was used to implement Pearson correlation analyses between ALFFs in 116 AAL brain areas and the indicators of GMERA. The ALFFs-difference of eyes-opened minus eyes-closed was used as an index of transition from internal feeling and self-consciousness to external stimulus processing (Nakano et al., 2012; Nakano, 2015; Zhang et al., 2020b; Zhang and Liu, 2021). Its significance was detected by correlation analyses between that and the indicators of GMERA. Since the original ALFF for each AAL brain area (the average ALFF of its all voxels) was extracted (Wang, 2009; Li W. S., 2015; Tang et al., 2018), a multiple comparisons correction could not be made for the correlation analyses mentioned above (Yin et al., 2020; Zhang et al., 2020b; Zhang and Liu, 2021). The relevant brain areas were visualized with the BrainNet Viewer (Xia et al., 2013)^1^.

## Results

SPSS 19.0 was used for statistics. There were 53 valid participants in PREMERT, 46 valid participants in the eyes-closed resting-state, and 51 valid participants in the eyes-opened resting-state because seven participants’ head movements were greater than 2 mm in the eyes-closed resting-state and two participants in the eyes-opened resting-state.

### Behavioral data

Each microexpression *M* was significantly greater than random level 1/6 with a single sample *t*-test (*p*s < 0.001). Each microexpression *SD* was significantly greater than random level 0 with a single sample *t*-test (*p*s < 0.001). They were shown in Table 1.

**TABLE 1:** The scores of PREMERT.

Microexpression	PREMERT *M* ± *SD* (*n* = 53)	*T*-value	Cohen’s *d*
Sadness *M*	0.32 ± 0.21	5.42***	0.73
Fear *M*	0.28 ± 0.15	5.55***	0.76
Anger *M*	0.56 ± 0.26	11.12***	1.51
Disgust *M*	0.49 ± 0.21	11.42***	1.54
Surprise *M*	0.68 ± 0.2	18.59***	2.57
Happiness *M*	0.78 ± 0.26	17.23***	2.36
Sadness *SD*	0.15 ± 0.06	17.65***	2.50
Fear *SD*	0.16 ± 0.06	18.70***	2.67
Anger *SD*	0.16 ± 0.06	19.89***	2.67
Disgust *SD*	0.17 ± 0.05	22.71***	3.40
Surprise *SD*	0.16 ± 0.08	15.60***	2.00
Happiness *SD*	0.10 ± 0.08	8.60***	1.25

***p < 0.001.

Exploratory factor analysis was conducted on the six microexpression *M*s (please refer to Materials and methods section). The results showed that KMO = 0.60 ≥ 0.5 and sphericity test *P* < 0.001, thus, it was suitable for exploratory factor analysis. Only one factor was extracted, whose initial eigen value > 1. It could explain 55.82% of the total variance. This common factor is the GMERA. The factor matrix was shown in Table 2.

**TABLE 2:** The exploratory factor analysis of microexpression recognition accuracy.

Microexpression	Factor
	
	GMERA
Happiness *M*	0.922
Anger *M*	0.723
Sadness *M*	0.709
Surprise *M*	0.691
Disgust *M*	0.560
Fear *M*	0.480

Extraction method: principal axis factor decomposition. One factor has been extracted. Eight iterations are required.

The four indicators such as GMERAL, GMERAF, GMERAB, and GMERABF are shown in Table 3. The GMERAL of PREMERT was significantly higher than the random level 1/6 but was not significantly different with 0.5. The GMERAF, GMERAB, and GMERABF were all significantly greater than the random level 0.

**TABLE 3:** The indicators of the GMERA.

Index	PREMERT (M ± SD)	*T*-value	Cohen’s *d*	EMERT1 (M ± SD)	*r*_PR–EM1_	EMERT2 (*M* ± *SD*)	*r*_PR–EM2_	JACBART	*r*_PR–J_	Expression	*r*_PR–E_
GMERAL	0.52 ± 0.16	15.93***	2.21	0.58 ± 0.15	0.757**	0.62 ± 0.15	0.836**	0.58 ± 0.17	0.904**	0.68 ± 0.18	0.590**
GMERAF	0.24 ± 0.07	25.55***	3.43	0.26 ± 0.07	0.570**	0.25 ± 0.07	0.510**	0.29 ± 0.09	–	0.23 ± 0.07	–
GMERAB	0.15 ± 0.03	37.76***	5	0.15 ± 0.04	–	0.14 ± 0.04	0.346*				
GMERABF	0.07 ± 0.02	21.78***	3.5	0.07 ± 0.03	0.413**	0.07 ± 0.02	–				

*r*_PR–J_ was the *r* between PREMERT and JACBART. *r*_PR–E_ was the *r* between PREMERT and expression. *r*_PR–EM1_ was the *r* between PREMERT and EMERT1. r_PR–EM2_ was the *r* between PREMERT and EMERT2.

*p < 0.05, **p < 0.01, and ***p < 0.001.

Kolmogorov–Smirnov analysis found that all four indicators of the GMERA in PREMERT followed a normal distribution, *p*s > 0.05. Pearson correlation analysis found that the GMERAL, GMERAF, GMERAB, and GMERABF in PREMERT were significantly positively correlated with some corresponding indicators in EMERT1 and EMERT2. The GMERAL in PREMERT was significantly positively correlated with the accuracy in JACBART and expression. Of course, the four indicators in PREMERT were not all correlated with the corresponding indicators in EMERT1, EMERT2, JACBART, or expression. Even if it was correlated, the correlation coefficient was not 1.

### Brain imaging data

**FIGURE 2:**
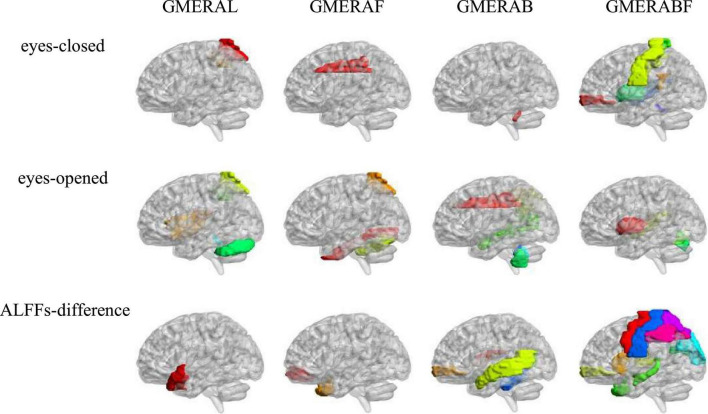
The ALFFs-relevant brain areas of the GMERA.

Pearson correlation analysis was made between ALFFs of the resting-state and the four indicators of the GMERA (refer to Table 4 and Figure 2; please refer to the Supplementary material for the original data).

**TABLE 4:** The *r*s between ALFFs of resting-state and the four indicators of the GMERA.

Resting-state	AAL brain area	ALFF (*M* ± *SD*)	GMERAL	GMERAF	GMERAB	GMERABF
Eyes-closed	Frontal_Mid_Orb_R	0.9046 ± 0.0702				−0.333*
Eyes-closed	Cingulum_Mid_L	0.9449 ± 0.0305		−0.299*		
Eyes-closed	Cingulum_Post_R	0.9358 ± 0.0445				−0.373*
Eyes-closed	Postcentral_L	0.841 ± 0.0602				0.349*
Eyes-closed	Postcentral_R	0.877 ± 0.0657				0.318*
Eyes-closed	Parietal_Sup_R	0.9339 ± 0.0607	0.332*			
Eyes-closed	Parietal_Inf_R	1.0619 ± 0.0626	0.389**			
Eyes-closed	Putamen_L	0.7931 ± 0.0376				−0.341*
Eyes-closed	Heschl_R	1.0802 ± 0.102				0.418**
Eyes-closed	Temporal_Sup_R	1.0748 ± 0.0659				0.416**
Eyes-closed	Vermis_1_2	1.8573 ± 0.3437				0.292*
Eyes-closed	Vermis_10	2.2163 ± 0.5643			0.298*	
Eyes-opened	Rolandic_Oper_R	0.8617 ± 0.0306	0.287*			
Eyes-opened	Insula_R	0.9707 ± 0.0383	0.300*			
Eyes-opened	Cingulum_Mid_R	0.9166 ± 0.0292			−0.390**	
Eyes-opened	Fusiform_R	0.87 ± 0.0273		−0.281*		
Eyes-opened	Parietal_Sup_R	0.9307 ± 0.0635	0.338*	0.282*		
Eyes-opened	Parietal_Inf_R	1.0503 ± 0.0555	0.329*		−0.351*	
Eyes-opened	Angular_R	1.0596 ± 0.0718			−0.295*	
Eyes-opened	Putamen_L	0.8082 ± 0.0314				−0.291*
Eyes-opened	Heschl_R	1.052 ± 0.0815				0.359**
Eyes-opened	Temporal_Sup_R	1.0466 ± 0.0538				0.320*
Eyes-opened	Temporal_Mid_R	0.9796 ± 0.0289			−0.283*	
Eyes-opened	Cerebelum_Crus1_L	0.9529 ± 0.0982	−0.306*			
Eyes-opened	Cerebelum_6_R	0.9167 ± 0.0491		−0.306*		
Eyes-opened	Cerebelum_9_L	0.7644 ± 0.2913			0.292*	
Eyes-opened	Vermis_1_2	1.8789 ± 0.37	0.287*			
Eyes-opened	Vermis_6	0.9287 ± 0.071				−0.326*
Eyes-opened	Vermis_7	0.8218 ± 0.0615				−0.297*
Eyes-opened	Vermis_9	1.076 ± 0.2724			0.391**	
Eyes-opened	Vermis_10	2.1888 ± 0.5697			0.330*	
ALFFs-difference	Precentral_L	−0.0177 ± 0.0322				−0.321*
ALFFs-difference	Precentral_R	−0.0314 ± 0.051				−0.368*
ALFFs-difference	Frontal_Mid_Orb_R	0.0226 ± 0.0401		−0.315*		
ALFFs-difference	Rolandic_Oper_R	−0.0038 ± 0.0192			−0.431**	
ALFFs-difference	Frontal_Mid_Orb_R	0.0215 ± 0.0445			0.321*	0.305*
ALFFs-difference	Hippocampus_L	0.0123 ± 0.0284				0.296*
ALFFs-difference	Cuneus_L	−0.0627 ± 0.1053				−0.293*
ALFFs-difference	Occipital_Sup_L	−0.0093 ± 0.0562				−0.306*
ALFFs-difference	Postcentral_L	−0.0395 ± 0.0437				−0.329*
ALFFs-difference	Postcentral_R	−0.0471 ± 0.047				−0.390**
ALFFs-difference	Parietal_Sup_L	−0.0054 ± 0.0499				−0.308*
ALFFs-difference	Parietal_Inf_L	−0.0094 ± 0.0343				−0.319*
ALFFs-difference	Parietal_Inf_R	−0.0103 ± 0.0484				−0.382**
ALFFs-difference	Caudate_L	0.0064 ± 0.0386				0.393**
ALFFs-difference	Thalamus_R	−0.0134 ± 0.0354				0.367*
ALFFs-difference	Temporal_Pole_Sup_L	0.0069 ± 0.0486	0.294*			
ALFFs-difference	Temporal_Mid_L	−0.0004 ± 0.0231			−0.376*	
ALFFs-difference	Temporal_Mid_R	−0.0086 ± 0.0267			−0.339*	
ALFFs-difference	Temporal_Pole_Mid_R	−0.0001 ± 0.022		−0.303*		0.375*
ALFFs-difference	Cerebelum_3_L	0.0345 ± 0.1515			0.336*	
ALFFs-difference	Cerebelum_3_R	0.027 ± 0.1784			0.368*	
ALFFs-difference	Cerebelum_4_5_L	−0.0108 ± 0.0515			0.342*	
ALFFs-difference	Vermis_3	0.0049 ± 0.1315			0.332*	

*p < 0.05 and **p < 0.01.

The GMERAL was positively related to some areas of the expression recognition network (Insula_R in the eyes-opened resting-state; and Temporal_Pole_Sup_L in the ALFFs-difference) (Hu et al., 2009; Yin et al., 2020; Zhang et al., 2020b), the microexpression consciousness and attention network (Rolandic_Oper_R and Insula_R in the eyes-opened resting-state; Parietal_Sup_R and Parietal_Inf_R in the eyes-closed and eyes-opened resting-states; and Temporal_Pole_Sup_L in the ALFFs-difference) (Dehaene et al., 2011; Huang et al., 2017; Yin et al., 2020; Zhang et al., 2020b,^2021^; Zhang and Liu, 2021), and the motor network for the change from expression backgrounds to microexpression (Rolandic_Oper_R and Vermis_1_2 in the eyes-opened resting-state) (Penhune and Steele, 2012; Zhang, 2014; Yin et al., 2020; Zhang et al., 2020b,^2021^; Zhang and Liu, 2021); but was negatively related to some area of the motor network (Cerebelum_Crus1_L in the eyes-opened resting-state).

The GMERAF was positively related to some areas of the microexpression consciousness and attention network (Parietal_Sup_R in the eyes-opened resting state); but was negatively related to some areas of the expression recognition network (Cingulum_Mid_L in the eyes-closed resting-state; Fusiform_R in the eyes-opened resting-state; and Temporal_Pole_Mid_R in the ALFFs-difference), the microexpression consciousness and attention network (Cingulum_Mid_L in the eyes-closed resting-state; Frontal_ Mid_Orb_R and Temporal_Pole_Mid_R in the ALFFs-difference), and the motor network for the change from expression backgrounds to microexpression (Cingulum_Mid_L in the eyes-closed resting-state; and Cerebelum_6_R in the eyes-opened resting-state).

The GMERAB was positively related to some areas of the microexpression consciousness and attention network (Frontal_Mid_Orb_R in the ALFFs-difference) and the motor network for the change from expression backgrounds to microexpression (Vermis_10 in the eyes-closed and eyes-opened resting-states; Cerebelum_9_L and Vermis_9 in the eyes-opened resting-states; and Cerebelum_3_L, Cerebelum_3_R, Cerebelum_4_5_L, and Vermis_3 in the ALFFs-difference); but was negatively related to some areas of the expression recognition network (Cingulum_Mid_R and Angular_R in the eyes-opened resting-state; Temporal_Mid_R in the eyes-opened resting-state and the ALFFs-difference; and Temporal_Mid_L in the ALFFs-difference), the microexpression consciousness and attention network (Rolandic_Oper_R in the ALFFs-difference; Cingulum_Mid_R, Parietal_Inf_R, and Angular_R in the eyes-opened resting-state; Temporal_Mid_R in the eyes-opened resting-state and the ALFFs-difference; and Temporal_Mid_L in the ALFFs-difference), and the motor network for the change from expression backgrounds to microexpression (Rolandic_Oper_R in the ALFFs-difference).

The GMERABF was positively related to some areas of the expression recognition network (Postcentral_L and Postcentral_R in the eyes-closed resting-state; Heschl_R and Temporal_Sup_R in the eyes-closed and eyes-opened resting-states; and Hippocampus_L, Temporal_Pole_Mid_R, and Thalamus_R in the ALFFs-difference), the microexpression consciousness and attention network (Heschl_R and Temporal_Sup_R in the eyes-closed and eyes-opened resting-states; and Frontal_Mid_Orb_R, Hippocampus_L, Temporal_Pole_Mid_R, Caudate_L, and Thalamus_R in the ALFFs-difference), and the motor network for the change from expression backgrounds to microexpression (Postcentral_L, Postcentral_R, and Vermis_1_2 in the eyes-closed resting-state; Thalamus_R in the ALFFs-difference); but was negatively related to some areas of the expression recognition network (Cingulum_Post_R in the eyes-closed resting-state; Postcentral_L, Postcentral_R, Cuneus_L, and Occipital_Sup_L in the ALFFs-difference), the microexpression consciousness and attention network (Cingulum_Post_R in the eyes-closed resting-state; and Precentral_L, Precentral_R, Frontal_Mid_Orb_R, Parietal_Sup_L, Parietal_Inf_L, Parietal_ Inf_R in the ALFFs-difference), and the motor network for the change from expression backgrounds to microexpression (Putamen_L, Vermis_6, and Vermis_7 in the eyes-opened resting-state; Precentral_L, Precentral_R, Postcentral_L, and Postcentral_R in the ALFFs-difference).

### Comparison of relevant brain areas between the general microexpression recognition ability and different types of microexpression recognition

**FIGURE 3:**
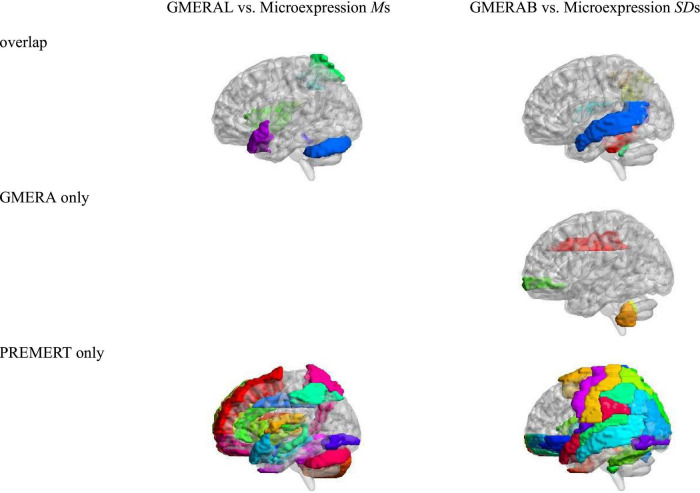
The overlap of relevant brain areas between GMERAL and Microexpression *M*s, and between GMERAB and Microexpression *SD*s.

Using the neuroimaging data from Zhang et al. (2020b), the relevant brain areas of the six microexpression *M*s were added up to form the relevant brain areas of the microexpression *M*s, and the relevant brain areas of the six microexpression *SD*s were added up to form the relevant brain areas of the microexpression *SD*s. We compared the relevant brain areas of the GMERAL in the current study with the microexpression *M*s in Zhang et al. (2020b), because they both measured the microexpression recognition ability, and we compared the relevant brain areas of the GMERAB in the current study with the microexpression *SD*s in Zhang et al. (2020b), because they both measured the microexpression recognition ability fluctuation by expression backgrounds, to get the consistency (particularity) of relevant brain areas between them (see Table 5 and Figure 3).

(1) There were nine common relevant brain areas between the GMERAL and the Microexpression *M*s, such as some areas of the expression recognition network (Insula_R and Temporal_Pole_Sup_L), the microexpression consciousness and attention network (Parietal_Sup_R, Parietal_Inf_R, Insula_R, and Temporal_Pole_Sup_L), and the motor network for the change from expression backgrounds to microexpression (Rolandic_Oper_R, Cerebelum_Crus1_L, and Vermis_1_2). There were 0 brain areas only related to the GMERAL. There were 51 brain areas only related to the Microexpression *M*s.
(2) There were 12 common relevant brain areas between the GMERAB and the Microexpression *SD*s, such as some areas of the expression recognition network (Temporal_ Mid_L and Temporal_Mid_R), the microexpression consciousness and attention network (Parietal_Inf_R, Angular_R, Temporal_Mid_L, and Temporal_Mid_R), and the motor network for the change from expression backgrounds to microexpression (Rolandic_Oper_R, Cerebelum_3_L, Cerebelum_3_R, Cerebelum_4_5_L, Vermis_3, and Vermis_10). There were four brain areas only related to the GMERAB, such as some areas of the microexpression consciousness and attention network (Frontal_Mid_Orb_R and Cingulum_Mid_R) and the motor network for the change from expression backgrounds to microexpression (Cerebelum_9_L and Vermis_9). There were 76 brain areas only related to the microexpression *M*s.

**TABLE 5:** The overlap of relevant brain areas between GMERAL and microexpression *M*s, and between GMERAB and microexpression *SD*s.

Resting state	AAL brain area	GMERAL	Microexpression *M*s	Overlap	Resting state	AAL brain area	GMERAB	Microexpression *SD*s	Overlap
Eyes-closed	Parietal_Sup_R	1	1	2	Eyes-closed	Vermis_10	1	1	2
Eyes-closed	Parietal_Inf_R	1	1	2	Eyes-opened	Parietal_Inf_R	1	1	2
Eyes-opened	Rolandic_Oper_R	1	1	2	Eyes-opened	Angular_R	1	1	2
Eyes-opened	Insula_R	1	1	2	Eyes-opened	Temporal_Mid_R	1	1	2
Eyes-opened	Parietal_Sup_R	1	1	2	Eyes-opened	Vermis_10	1	1	2
Eyes-opened	Parietal_Inf_R	1	1	2	Difference	Rolandic_Oper_R	1	1	2
Eyes-opened	Cerebelum_Crus1_L	1	1	2	Difference	Temporal_Mid_L	1	1	2
Eyes-opened	Vermis_1_2	1	1	2	Difference	Temporal_Mid_R	1	1	2
Difference	Temporal_Pole_Sup_L	1	1	2	Difference	Cerebelum_3_L	1	1	2
Eyes-closed	Frontal_Sup_L	0	1	1	Difference	Cerebelum_3_R	1	1	2
Eyes-closed	Rolandic_Oper_R	0	1	1	Difference	Cerebelum_4_5_L	1	1	2
Eyes-closed	Frontal_Sup_Medial_L	0	1	1	Difference	Vermis_3	1	1	2
Eyes-closed	Frontal_Sup_Medial_R	0	1	1	Eyes-opened	Cingulum_Mid_R	1	0	1
Eyes-closed	Insula_R	0	1	1	Eyes-opened	Cerebelum_9_L	1	0	1
Eyes-closed	Cingulum_Ant_L	0	1	1	Eyes-opened	Vermis_9	1	0	1
Eyes-closed	Cingulum_Mid_L	0	1	1	Difference	Frontal_Mid_Orb_R	1	0	1
Eyes-closed	Amygdala_L	0	1	1	Eyes-closed	Frontal_Sup_Orb_R	0	1	1
Eyes-closed	Fusiform_R	0	1	1	Eyes-closed	Rolandic_Oper_R	0	1	1
Eyes-closed	Parietal_Sup_L	0	1	1	Eyes-closed	Frontal_Mid_Orb_L	0	1	1
Eyes-closed	Precuneus_L	0	1	1	Eyes-closed	Hippocampus_R	0	1	1
Eyes-closed	Thalamus_L	0	1	1	Eyes-closed	Cuneus_L	0	1	1
Eyes-closed	Thalamus_R	0	1	1	Eyes-closed	Occipital_Mid_L	0	1	1
Eyes-closed	Heschl_L	0	1	1	Eyes-closed	Postcentral_R	0	1	1
Eyes-closed	Heschl_R	0	1	1	Eyes-closed	Parietal_Inf_R	0	1	1
Eyes-closed	Cerebelum_Crus1_L	0	1	1	Eyes-closed	Angular_R	0	1	1
Eyes-closed	Cerebelum_3_L	0	1	1	Eyes-closed	Caudate_L	0	1	1
Eyes-closed	Cerebelum_4_5_L	0	1	1	Eyes-closed	Caudate_R	0	1	1
Eyes-closed	Vermis_3	0	1	1	Eyes-closed	Putamen_L	0	1	1
Eyes-opened	Frontal_Sup_Orb_L	0	1	1	Eyes-closed	Pallidum_L	0	1	1
Eyes-opened	Frontal_Sup_Orb_R	0	1	1	Eyes-closed	Heschl_R	0	1	1
Eyes-opened	Rolandic_Oper_L	0	1	1	Eyes-closed	Temporal_Sup_L	0	1	1
Eyes-opened	Insula_L	0	1	1	Eyes-closed	Temporal_Mid_L	0	1	1
Eyes-opened	Cingulum_Ant_L	0	1	1	Eyes-closed	Vermis_1_2	0	1	1
Eyes-opened	Hippocampus_L	0	1	1	Eyes-closed	Vermis_3	0	1	1
Eyes-opened	ParaHippocampal_L	0	1	1	Eyes-opened	Precentral_L	0	1	1
Eyes-opened	Amygdala_L	0	1	1	Eyes-opened	Precentral_R	0	1	1
Eyes-opened	Occipital_Inf_L	0	1	1	Eyes-opened	Frontal_Sup_Orb_R	0	1	1
Eyes-opened	Fusiform_R	0	1	1	Eyes-opened	Supp_Motor_Area_L	0	1	1
Eyes-opened	Precuneus_L	0	1	1	Eyes-opened	Frontal_Mid_Orb_R	0	1	1
Eyes-opened	Caudate_L	0	1	1	Eyes-opened	Insula_R	0	1	1
Eyes-opened	Thalamus_L	0	1	1	Eyes-opened	Hippocampus_R	0	1	1
Eyes-opened	Thalamus_R	0	1	1	Eyes-opened	Lingual_R	0	1	1
Eyes-opened	Heschl_L	0	1	1	Eyes-opened	Occipital_Mid_L	0	1	1
Eyes-opened	Heschl_R	0	1	1	Eyes-opened	Occipital_Mid_R	0	1	1
Eyes-opened	Temporal_Pole_Sup_L	0	1	1	Eyes-opened	Occipital_Inf_L	0	1	1
Eyes-opened	Temporal_Pole_Sup_R	0	1	1	Eyes-opened	Occipital_Inf_R	0	1	1
Eyes-opened	Cerebelum_3_L	0	1	1	Eyes-opened	Fusiform_R	0	1	1
Eyes-opened	Cerebelum_4_5_L	0	1	1	Eyes-opened	Postcentral_L	0	1	1
Eyes-opened	Cerebelum_6_R	0	1	1	Eyes-opened	Parietal_Sup_L	0	1	1
Eyes-opened	Vermis_3	0	1	1	Eyes-opened	Parietal_Sup_R	0	1	1
Difference	Frontal_Inf_Tri_R	0	1	1	Eyes-opened	Parietal_Inf_L	0	1	1
Difference	Insula_L	0	1	1	Eyes-opened	Angular_L	0	1	1
Difference	Insula_R	0	1	1	Eyes-opened	Caudate_L	0	1	1
Difference	Parietal_Inf_L	0	1	1	Eyes-opened	Caudate_R	0	1	1
Difference	Putamen_R	0	1	1	Eyes-opened	Pallidum_L	0	1	1
Difference	Heschl_R	0	1	1	Eyes-opened	Pallidum_R	0	1	1
Difference	Temporal_Pole_Sup_R	0	1	1	Eyes-opened	Vermis_1_2	0	1	1
Difference	Temporal_Inf_R	0	1	1	Eyes-opened	Vermis_3	0	1	1
Difference	Cerebelum_Crus1_L	0	1	1	Eyes-opened	Vermis_4_5	0	1	1
Difference	Cerebelum_Crus2_R	0	1	1	Eyes-opened	Vermis_7	0	1	1
					Difference	Frontal_Sup_Orb_L	0	1	1
					Difference	Frontal_Mid_Orb_L	0	1	1
					Difference	Frontal_Inf_Orb_L	0	1	1
					Difference	Rolandic_Oper_L	0	1	1
					Difference	Olfactory_L	0	1	1
					Difference	Hippocampus_R	0	1	1
					Difference	Amygdala_R	0	1	1
					Difference	Cuneus_L	0	1	1
					Difference	Cuneus_R	0	1	1
					Difference	Occipital_Sup_L	0	1	1
					Difference	Occipital_Sup_R	0	1	1
					Difference	Occipital_Mid_L	0	1	1
					Difference	Occipital_Mid_R	0	1	1
					Difference	Occipital_Inf_L	0	1	1
					Difference	Occipital_Inf_R	0	1	1
					Difference	SupraMarginal_L	0	1	1
					Difference	SupraMarginal_R	0	1	1
					Difference	Angular_R	0	1	1
					Difference	Caudate_L	0	1	1
					Difference	Caudate_R	0	1	1
					Difference	Putamen_L	0	1	1
					Difference	Putamen_R	0	1	1
					Difference	Thalamus_L	0	1	1
					Difference	Thalamus_R	0	1	1
					Difference	Temporal_Sup_R	0	1	1
					Difference	Temporal_Pole_Sup_L	0	1	1
					Difference	Temporal_Inf_R	0	1	1
					Difference	Cerebelum_4_5_R	0	1	1
					Difference	Cerebelum_6_L	0	1	1
					Difference	Cerebelum_10_L	0	1	1
					Difference	Vermis_4_5	0	1	1
					Difference	Vermis_7	0	1	1

If an AAL brain area was related to the GMERAL, GMERAB, one of the microexpression *M*s, or one of the microexpression *SD*s, the corresponding number was 1, else, the corresponding number was 0. The numbers of the GMERA and PREMERT added up to form the number of the overlap, such as two indicating the AAL brain area was related to both the GMERA and PREMERT and 1 indicating the AAL brain area was related to one of the GMERA and PREMERT. The relevant brain areas of microexpression *M*s and microexpression *SD*s in PREMERT were derived from Zhang et al. (2020b).

## Discussion

### The general microexpression recognition ability in PREMERT had good reliability and validity

In PREMERT, a common factor GMERA was extracted from the recognition accuracy rates of six microexpression types by exploratory factor analysis. Four indicators were calculated from six microexpression *M*s and six microexpression *SD*s, such as GMERAL, GMERAF, GMERAB, and GMERABF. The GMERAL was significantly higher than the random level 1/6, but was not significantly different with 0.5, indicating that the participants had certain general microexpression recognition abilities. The GMERAF, GMERAB, and GMERABF were all significantly greater than the random level 0, indicating that the GMERA fluctuated, and there were background effect and background effect fluctuations.

The GMERAL, GMERAF, GMERAB, and GMERABF in PREMERT were significantly positively correlated with some corresponding indicators in EMERT1 and EMERT2, indicating that the four indicators of the GMERA had good parallel-forms reliability and calibration validity. The GMERAL in PREMERT was significantly positively correlated with the correct rate in JACBART and expression, indicating that the GMERAL of PREMERT had good calibration validity. Of course, the four indicators of the GMERA in PREMERT were not all correlated with the corresponding indicators in EMERT1, EMERT2, JACBART, or expression. Even if it was correlated, the correlation coefficient was not 1, indicating that the GMERA in PREMERT had ecological validity and was indeed different from those in EMERT1, EMERT2, JACBART, and expression.

Because the pseudo-random design in PREMERT ensured that each participant took the same test, the GMERAL could measure the GMERA level of each participant, including the recognition ability of six microexpression types in seven expression backgrounds. The GMERAF measures the GMERA fluctuation degree (stability) among microexpression types. The GMERAB measures the overall GMERA background effect, that is, the degree of fluctuation (stability) in different expression backgrounds. The GMERABF measures the degree of fluctuation (stability) of the GMERA background effect among microexpression types. In Zhang et al. (2020b), each microexpression type was recognized under seven different expression backgrounds (sadness, fear, anger, disgust, surprise, happiness, and neutral), thus, there were six microexpression recognition accuracy rates for a microexpression type (except a microexpression under the expression background of its own emotion, in that case, the microexpression became a normal expression), which were the third layer in the three-layer hierarchical structure of human microexpression recognition ability. Microexpression *M* was used to summarize a microexpression type recognition under seven expression backgrounds, and microexpression *SD* was used to generalize a microexpression type recognition fluctuation (stability) among seven expression backgrounds. Thus, microexpression *M* and microexpression *SD* were the second layers generalized from the third layer. The four indicators of the GMERA were the highest layer generalized from the second layer to provide a concise and comprehensive overview of key information of PREMERT. Since the GMERA could be obtained with good reliability and validity in PREMERT, PREMERT was proved a good test to measure the GMERA in turn.

In the third layer, there was only one indicator, the microexpression recognition accuracy, but it contained 7 (expression backgrounds: neutral vs. sadness vs. fear vs. anger vs. disgust vs. surprise vs. happiness) × 6 (microexpressions: sadness vs. fear vs. anger vs. disgust vs. surprise vs. happiness) = 42 different sub-indicators (Zhang et al., 2017; Zhu et al., 2017; Yin et al., 2019). Those were too many to generalize an individual’s microexpression recognition ability. In the second layer, two indicators such as microexpression *M* and microexpression *SD* were set up to get a concise and comprehensive overview of the third layer (Yin et al., 2020; Zhang et al., 2020b). The indicator number in the second layer was twice as much as that in the third layer, but it only contained 2 (indicators: microexpression *M* vs. microexpression *SD*) × 6 (microexpressions: sadness vs. fear vs. anger vs. disgust vs. surprise vs. happiness) = 12 different sub-indicators. However, those were still too many to generalize an individual’s microexpression recognition ability. Therefore, the current study put forward four indicators of the GMERA in the highest layer such as the GMERAL, GMERAF, GMERAB, and GMERABF to get a concise and comprehensive overview of the second layer. The indicator number in the highest layer was also twice as much as that in the second layer, but it only contained four different sub-indicators. We can see that if we want to reduce the sub-indicators and keep as much information as possible about the lower layer to get a concise and comprehensive overview, the indicators of the higher layer have to be twice the lower layer. When the indicators and their sub-indicators overlapped, the highest layer was received with convergence. The Stepwise Higher-Order Factor Analyses tend to find a common factor to get the most concise overview but lose much information, which is a defect. However, the GIF (Vernon, 1966) and GPF (Musek, 2007) did not consider that defect, so they lost a lot of information, especially the ability fluctuation. The current study pointed out and considered that defect, and used the indicators twice to avoid losing key information on the lower layer. This approach can be generalized to other areas of ability including GIF and GPF in the future.

### The relevant resting-state brain areas of the general microexpression recognition ability in PREMERT

The amplitude of low-frequency fluctuations of some brain areas in both eyes-closed and eyes-opened resting-states and their ALFFs-difference could predict the four indicators of the GMERA in the expression recognition network, the microexpression consciousness and attention network, and the motor network for the change from expression backgrounds to microexpression. The three cognitive processes of microexpression recognition were logically deduced, and then the relevant brain areas were classified into the three cognitive processes according to the functions of these brain areas found in previous studies (Yin et al., 2020; Zhang et al., 2020b). Logically speaking, if the functions of the relevant brain areas for the three cognitive processes are strong, then the level of the general microexpression recognition ability should be high, which was partly proved by the positively relevant brain areas of the GMERAL. The negatively relevant brain area of the GMERAL was some area of the motor network, which might be because of good motor function, and the participants could easily switch between the expression backgrounds and the microexpression, thus it was difficult for them to get consciousness of the new stimulus of microexpression (Huang et al., 2015; Zhang et al., 2015).

If we improve an individual’s function of the positively relevant brain areas of GMERAL, and/or reduce his/her function of the negatively relevant brain areas of GMERAL, his/her GMERA level may improve. This is a good thing. However, if we improve an individual’s function of the positively relevant brain areas of GMERAF, GMERAB, or GMERABF, and/or reduce his/her function of the negatively relevant brain areas of GMERAF, GMERAB, or GMERABF, his/her GMERA fluctuation, GMERA background effect, or GMERAB fluctuation may improve. This is a mixed blessing, which may improve some special microexpression recognition, but can reduce GMERA stability.

The GMERAF, GMERAB, and GMERABF measured the GMERA fluctuation caused by the microexpression emotional types and expression backgrounds. Therefore, logically speaking, if the functions of some relevant brain areas for the three cognitive processes are strong, the participants will recognize each microexpression with each emotional type and under each expression background well to make the recognition stable, and the GMERA fluctuation should be low, which was partly proved by the negatively relevant brain areas of the GMERAF, GMERAB, and GMERABF. Of course, if the functions of other relevant brain areas for the three cognitive processes are strong, the participants may sensitively recognize expression types of both microexpressions and expression backgrounds, and easily get consciousness and attention on both microexpressions and expression backgrounds, thus, they will be strongly influenced by microexpression emotional types and expression backgrounds; they may also easily change from expression backgrounds to microexpression and from microexpression to expression backgrounds, thus it was difficult for them to get consciousness of the novel stimulus of microexpression (Huang et al., 2015; Zhang et al., 2016). These should make the GMERA fluctuation high, which was partly proved by the positively relevant brain areas of the GMERAF, GMERAB, and GMERABF.

Yin et al. (2020) found that ALFFs of some brain areas in a resting state were related to the six microexpression types recognition in EMERT. Zhang et al. (2020b) found that ALFFs of some brain areas in a resting-state were related to the six microexpression types recognition in PREMERT. But they did not put forward or explore the GMERA. The current study found a kind of inherited neural activation pattern indicated by specific resting-state activations that could predict the GMERA, which indicated that the spontaneous brain activity in the resting-state is a stable index to measure microexpression perception performance, and could in turn prove that the GMERA is an individual cognitive characteristic from brain mechanisms since the spontaneous brain activity in the resting state is a stable index to measure the individual cognitive characteristics (Heuvel and Pol, 2010; Li et al., 2016; Liu et al., 2017; Jiang et al., 2018). In the future, task-state fMRI or event-related potential ERP experiments are needed to further explore the specific functions of each brain area in the GMERA cognitive processes.

### The overlap and difference of relevant brain areas between the general microexpression recognition ability and different types of microexpression recognition

In the current study, the GMERAL measured the level of the GMERA, and the GMERAB measured the background effect of the GMERA, which was the highest layer. In Zhang et al. (2020b), the microexpression *M*s measured the individual’s microexpression recognition ability under different expression backgrounds, and the microexpression *SD*s measured the individual’s microexpression recognition ability fluctuation by different expression backgrounds, which were the second layers. We compared the relevant brain areas of the highest layer with the second layer to get the overlap and difference of relevant brain areas between them and found that they all belong to the expression recognition network, the microexpression consciousness and attention network, and the motor network for the change from expression backgrounds to microexpression, but the relevant brain areas of GMERAL and GMERAB were concise and critical, and the relevant brain areas of microexpression *M*s and microexpression *SD*s were rich and tedious. Therefore, the GMERA was much more focused and simplified to measure the microexpression recognition ability and its fluctuation in the relevant brain areas, which indicated that the relevant brain areas of the GMERA should be the “higher-order” areas from innate heredity and influenced by learning compared to the brain areas related to different types of microexpression recognition. This is beneficial to improve the function of these critical brain areas to develop the microexpression recognition ability in the future because the more the areas of the brain areas need intervention, the more work intervention, and vice versa.

### The limitations of the current study

The participants had no training in lie detection, microexpression recognition, or other detection skills. But their cognitive skill levels were not measured. They had similar Chinese college entrance examination scores, which could not make sure that they had similar IQs. Despite this, it was explored which brain activity differences would influence differences in microexpression recognition ability. Therefore, individual differences were not interfering factors but beneficial factors in the current study. However, it is very valuable to explore how IQ and other cognitive skill levels influence GMERA in the future. The spontaneous brain activity in the resting state was impacted by many individual cognitive characteristics (Liu et al., 2017), such as emotional ability (Heuvel and Pol, 2010; Li X., 2015; Xue et al., 2015), IQ (Langer et al., 2012; Zhang, 2021), creativity (Li et al., 2016; Jiang et al., 2018), music skills (Leipold et al., 2021; Long, 2022), and personality (Adelstein et al., 2011; Wang, 2016). These individual cognitive characteristics may affect the GMERA by influencing spontaneous brain activity in the resting state. For example, some frontal and parietal lobes were the commonly relevant brain areas between IQ and GMERA. Of course, the GMERA had its unique brain areas. In the future, we can compare the brain areas related to these cognitive characteristics revealed by different literature and the GMERA revealed by the current study explores the qualitative relationship among them. We should measure these cognitive characteristics, the GMERA, and the spontaneous brain activity in the resting state of the same group of participants, and then use correlation, path analysis, structural equation model, and other methods to investigate the quantitative relationship among them.

At the time of recruitment, participants were required to be right-handed only to participate in the current study. It was judged whether a participant was right-handed or not by asking which hand he/she used to carry out various activities in daily life, such as writing, eating with chopsticks, and carrying things. It was further verified that the participant signed informed consent. Since a formal handedness test was not performed, there might be some participants writing with the right hand but being left-handers. Although the likelihood of this is low, it should be considered and excluded in future studies.

The participants were asked to close their eyes or look naturally without great concentration at a cross on a screen with their eyes opened, take a break for 8 min each, and try not to think about anything or something intently (Li et al., 2016; Liu et al., 2017; Jiang et al., 2018; Zhang et al., 2020a; Zhang and Liu, 2021). The instruction seemed a bit weird because it is almost impossible for human beings to think of nothing. However, it is useful to make the participants think as less as possible because the resting state is to measure spontaneous brain activity without any cognitive activation from external stimuli or internal thinking. It was to keep the participants from focusing so much on the cross that it affected their resting state by asking the participants to look naturally without great concentration at the cross. Of course, this allowed visual information about the cross and things around to enter the participants’ brains, but this setting was necessary to simulate the effect of the visual background of the environment when the participants were at rest with their eyes opened. The instruction and setting method need to be further optimized to get a more desirable resting state.

The resting-state measurement is not without problems. Dizziness, stress, anxiety, sleepiness, etc., strongly influence resting-state activity (Heinrich et al., 2014; Mutschler et al., 2014). The current study instructed that the fMRI was not harmful, that participants should get enough sleep the night before and take it easy in the scan, and that claustrophobic people should not participate. These methods could reduce the distractions to some extent, but could not eliminate them completely. In addition, resting-state fMRI maybe not be a proper resting-state measure since the measurement is loud and partly even annoying (tight bore, etc.). Therefore, it is necessary to make the fMRI scan more comfortable and use EEG (electroencephalogram) to record the resting state.

What does resting-state activity really tell us about the neural underpinnings? Is it either a stable index of individual cognitive characteristics (Heuvel and Pol, 2010; Li et al., 2016; Liu et al., 2017; Jiang et al., 2018) or temporary emotional and cognitive states (Heinrich et al., 2014; Mutschler et al., 2014)? The participants took part in the resting-state fMRI scans a month before the PREMERT, and resting-state activity was still related to the GMERA in the PREMERT, which indicated that resting-state activity could predict the GMERA stably rather than temporarily, and it was a stable index of the individual microexpression recognition ability. Although the current study could not eliminate temporary emotional and cognitive states completely, the results showed that the temporary emotional and cognitive states a month ago could predict the GMERA, which indicated to some extent that the temporary emotional and cognitive states should be the stable individual cognitive characteristics induced and measured by the resting-state fMRI scans, that means when a participant takes part in the resting-state fMRI scans, he/she will produce the specific temporary emotional and cognitive states each time, and different participants will produce different specific temporary emotional and cognitive states in the same time. That may be caused by the brain function difference between heredity and learning. If we can eliminate temporary emotional and cognitive states as completely as possible (as mentioned above), more relevant brain areas of the GMERA may be found. Those possibilities can be investigated in the future.

## Conclusion

The current study put forward and established the GMERA, and explored the relevant resting-state brain activity of its indicators. The results showed the following:

(1) There was a three-layer hierarchical structure in human microexpression recognition ability: The GMERA (the highest layer); recognition of a type of microexpression under different expression backgrounds (the second layer); and recognition of a certain microexpression under a certain expression background (the third layer). A common factor GMERA was extracted from the six microexpressions recognition in PREMERT. Four indicators of the GMERA were calculated from six microexpression *M*s and six microexpression *SD*s, such as GMERAL, GMERAF, GMERAB, and GMERABF, which had good parallel-forms reliability, calibration validity, and ecological validity. The GMERA provided a concise and comprehensive overview of the individual’s microexpression recognition ability. The PREMERT was proved as a good test to measure the GMERA.
(2) Amplitude of low-frequency fluctuations in both eyes-closed and eyes-opened resting-states and ALFFs-difference could predict the four indicators of the GMERA. The relevant resting-state brain areas were some areas of the expression recognition network, the microexpression consciousness and attention network, and the motor network for the change from expression backgrounds to microexpression.
(3) The relevant brain areas of the GMERA and different types of microexpression recognition belonged to the three cognitive processes, but the relevant brain areas of the GMERA were the “higher-order” areas to be more concise and critical than those of different types of microexpression recognition.

## Data availability statement

The original contributions presented in this study are included in the article/Supplementary material, further inquiries can be directed to the corresponding authors.

## Ethics statement

The studies involving human participants were reviewed and approved by the Scientific Review Committee of Faculty of Psychology, Southwest University, China. The patients/participants provided their written informed consent to participate in this study. Written informed consent was obtained from the individual(s) for the publication of any potentially identifiable images or data included in this article.

## Author contributions

JZ provided research ideas and financial support, responsible for research design, data collection and analysis, and manuscript writing. MY provided part of the research ideas, responsible for literature review, data analysis, and manuscript modification. DS was responsible for experimental programming and instrument provision. DL was responsible for guiding the design, implementation, data analysis and manuscript review, and provided financial support. All authors contributed to the article and approved the submitted version.

## Footnotes

1
http://www.nitrc.org/projects/bnv/
